# The Importance of Olfaction for Mixed Paternity in Birds

**DOI:** 10.1002/ece3.70863

**Published:** 2025-01-16

**Authors:** Charlotta Kvarnemo, Alice Anderstedt, Maria Strandh, Donald Blomqvist

**Affiliations:** ^1^ Department of Biological and Environmental Sciences University of Gothenburg Gothenburg Sweden; ^2^ Department of Biology Lund University Lund Sweden

**Keywords:** extra‐pair paternity, genetic compatibility, mate choice, monogamy, odour, promiscuity

## Abstract

Olfaction can aid individuals in finding genetically compatible mates in many animals, while high levels of mixed paternity may result from a limited ability to evaluate their mate's genetic profile against their own before mating. To test this suggestion and explore if olfaction may indeed influence mating patterns in birds, we combined published measures of olfactory ability with data on genetic mating pattern in the same species, across a phylogenetically broad range of species. We used three measures of olfaction: (1) olfactory bulb diameter, (2) olfactory bulb volume and (3) number of olfactory receptor genes (148, 134 and 48 species, respectively). These measures were then matched to species‐specific estimates of mating pattern, measured as percentage of broods with mixed paternity (> 1 male siring offspring in the same brood). Limited overlaps between the datasets resulted in 30 matched species for olfactory bulb diameter, 31 for olfactory bulb volume and 15 for olfactory receptor genes. Controlling for brain size (telencephalon), we then correlated olfaction to mating pattern, and found that the bigger the relative olfactory bulb diameter, the lower the proportion of mixed paternity. In contrast, there was no significant correlation between olfactory bulb volume or number of receptor genes and paternity. This study thus indicates that mating patterns in birds may be influenced by olfactory ability, measured as olfactory bulb diameter. Next, we suggest expanding the datasets by collecting olfactory‐focused measures, targeting species for which paternity measures already exist, to allow a full phylogenetic analysis.

## Graphical abstract

Olfaction can help individuals finding genetically compatible mates in many animals, while high levels of mixed paternity may reflect a reduced ability to evaluate a mate's profile against their own before mating. We tested this hypothesis by matching estimates of olfactory ability with mixed paternity for a broad range of bird species. As predicted, we found that the bigger the relative olfactory bulb diameter, the lower the percentage of broods with mixed paternity. However, we found no correlation between two other measures of olfactory ability and mixed paternity.

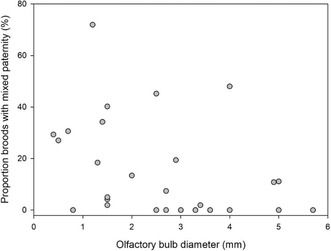

## 1 Introduction

Olfactory cues are important for mate choice and pair maintenance in a wide range of animals (Johansson and Jones 2007), with examples found in mammals (Petrulis 2013), lizards (Olsson et al. 2003), fish (Keller‐Costa, Canário, and Hubbard 2015; Lehtonen and Kvarnemo 2015; Lin et al. 2021) and birds (Caro, Balthazart, and Bonadonna 2015).

In many animals, offspring fitness is affected by the combination of the genes of their parents (or "how well the genes of the parents function together in their offspring"; Puurtinen, Ketola, and Kotiaho 2005) (Tregenza and Wedell 2000; Puurtinen, Ketola, and Kotiaho 2009). To be able to identify well‐matched mates, and avoid mating with poorly matched ones, one would expect discrimination before mating to evolve; however, such mate choice is rarely found (Jennions and Petrie 2000; Tregenza and Wedell 2000; Mays and Hill 2004; Puurtinen, Ketola, and Kotiaho 2005). One explanation is that genetic compatibility is often determined *after* mating, for example, through gamete recognition proteins, sperm selection or differential embryo mortality (Kosman and Levitan 2014). That said, some important aspects of genetic compatibility, especially those of complementary genes of the major histocompatibility complex (MHC), can be determined *before* mating using olfaction (Milinski 2006; Schubert, Nichols, and Winternitz 2021). This has been shown for example in fish, mammals, lizards and birds (e.g., Penn and Potts 1999; Penn 2002; Zelano and Edwards 2002; Olsson et al. 2003; Milinski et al. 2005; Havlicek and Roberts 2009; Lenz et al. 2009; Jaworska et al. 2017; Santos et al. 2018). The recognition mechanism involves olfactory cues, presumably in the form of volatiles or peptides that are produced by microbiota of the potential mate and compared against a self‐referenced odour (Schubert, Nichols, and Winternitz 2021). Many studies that have reported MHC‐dependent mate choice found that the choosy sex prefers a partner with somewhat dissimilar MHC alleles (reviewed by Milinski 2006).

Mating patterns in birds vary widely, and while mixed paternity is rare in some, it is common in others (e.g., Griffith, Owens, and Thuman 2002). Since males usually do not provide care for offspring that they sire in other males' nests, they should benefit from extra‐pair matings (i.e., matings outside the social pair bond). In contrast, it is more difficult to explain what female birds would gain from such matings (e.g., Brouwer and Griffith 2019). Despite decades of research, the underlying explanation for these broad scale differences is still unknown (Brouwer and Griffith 2019). However, differences between taxa in olfaction, together with genetic benefits of mate choice, might provide such an explanation. As elaborated below, we offer a hypothesis that species with well‐developed olfaction are able to identify a genetically compatible partner before mating. Both sexes should benefit from reproducing together with a compatible partner, so once they have found one, we expect them to remain ‘faithful’, resulting in low levels of mixed paternity in such taxa.

While some studies have been unable to find a link between extra‐pair matings and genetic benefits, two aspects of genetic compatibility have frequently been associated with extra‐pair matings: a high genetic similarity or relatedness between social mates, and a difference in heterozygosity between within‐pair and extra‐pair offspring (meta‐analysis: Arct, Drobniak, and Cichoń 2015; review: Brouwer and Griffith 2019; and references therein). Arct, Drobniak, and Cichoń (2015) even suggested that the failure of some studies to detect a positive relationship between the relatedness of social mates and the occurrence of extra‐pair young may have methodological causes.

Many passerine birds (songbirds) show social monogamy but high levels of mixed paternity (e.g., Griffith, Owens, and Thuman 2002). Passerines use olfaction in many contexts (e.g., Whittaker et al. 2011; Grieves et al. 2019; Van Huynh and Rice 2019; Song et al. 2021). However, they have small olfactory bulbs in relation to the size of their brain, compared to many other orders of birds, indicating less developed olfactory capabilities (Stager 1967). It has been suggested that high levels of mixed paternity are due to limited ability to evaluate their mate's genetic profile against their own before mating (Griffith and Immler 2009). In addition to differences in life history (Arnold and Owens 2002), such reduced ability may explain why mixed paternity is found much more frequently in passerines than in non‐passerines (e.g., Brouwer and Griffith 2019), assuming that extra‐pair matings allow poor matches to be corrected at the gamete level (Kosman and Levitan 2014). Several studies report higher fitness in extra‐pair young than in within‐pair young, although some have been unable to detect a difference (e.g., Griffith, Owens, and Thuman 2002; Bowers et al. 2015; and references therein).

At the other end of the spectrum, procellariiform seabirds are well‐known for their olfactory ability (Nevitt 2008). In concert with that, they have on average more than three times larger olfactory bulbs than passerines, adjusted for brain size (8.8 in passerines and 29.7 in procellariiform birds, expressed as a ratio between the longest diameter of the olfactory bulb and the longest diameter of the cerebral hemisphere, multiplied by 100; Zelenitsky et al. 2011). Long‐term genetic monogamy is common in these long‐lived procellariiforms (Bried, Pontier, and Jouventin 2003), and MHC‐based mate choice has been found at least in one species, the blue petrel *Halobaena caerulea* (Strandh et al. 2012). This ability may result in procellariiform seabirds forming pair bonds with well‐matched mates, resulting in fit offspring, and a mate choice that does not require correction through extra‐pair matings.

Olfaction has evolved for various ecological reasons, primarily related to habitat (semi‐aquatic species have relatively larger olfactory bulbs compared to terrestrial and aquatic species; Corfield et al. 2015). Here, our interest is whether the existing olfactory abilities are also associated with the species‐specific mating patterns. Given that olfaction is an important sensory mode to gain information in relation to mate choice, especially regarding genetic compatibility of potential mates, we predict that taxa with well‐developed capacity for olfaction are better equipped for making an accurate mate choice, thus facilitating long‐term reproductive (‘genetic’) monogamy, compared to taxa with less developed capacity for olfaction. In the latter taxa, an erroneous initial mate choice may need to be corrected at a later stage, for example, through extra‐pair fertilisations.

Olfactory ability can be assessed through the size (diameter or volume) of the olfactory bulb in the brain relative to the size of the rest of the brain. Olfactory bulb size varies greatly among avian taxa (Bang and Cobb 1968; Zelenitsky et al. 2011). Olfactory receptor (OR) genes encode odour perception and the total number of OR genes carried by a species offers another way to estimate olfactory ability (Steiger et al. 2008; Yohe et al. 2020; Trimmer et al. 2019). Number of OR genes is evolutionarily variable among tetrapods (Yohe et al. 2020) including birds (Steiger et al. 2008; Khan et al. 2015; Driver and Balakrishnan 2021).

Here, we cover a phylogenetically broad range of bird species and match measures of olfactory ability with measures of genetic mating pattern in the same species. We use three different measures of olfactory ability: (1) the diameter of the olfactory bulb (controlled for brain size), (2) the volume of the olfactory bulb (controlled for brain size) and (3) the number of OR genes for each species, and we correlate these measures to species‐specific estimates of reproductive monogamy, measured as percentage of broods with mixed paternity (i.e., more than one male siring offspring in the same brood). We expect species that are able to identify a suitable mate before mating via olfaction (based on measures 1, 2 or 3) to be more likely to show a high degree of reproductive monogamy (thus, a low value of mixed paternity), compared to species whose ability is less well developed.

## 2 Materials and Methods

This study is based on published data on olfactory bulb diameter (Bang and Cobb 1968; Bang 1971), olfactory bulb volume (Corfield et al. 2015) and number of OR genes (Steiger et al. 2008; Khan et al. 2015). We refer to these data sets as measures 1, 2 and 3, which contain data from 148, 134 and 48 species of birds, respectively.

The three data sets were matched against a data base of published studies of within‐ and extra‐pair paternity estimates, covering 401 species of birds in total (Valcu, Valcu, and Kempenaers 2021). When data were available from multiple studies of the same species, we calculated a species‐specific index of extra‐pair paternity by pooling the estimates from these studies. In addition, to search for papers published after 2021 and to collate data on mixed paternity from species that were excluded by Valcu, Valcu, and Kempenaers (2021), we carried out a literature search in Web of Science, using ‘extrapair copulation’, ‘extrapair paternity’, ‘mixed paternity’, ‘multiple paternity’, ‘paternity’ and ‘species name’ (common or scientific) as our search terms, for all previously unmatched species in sets 1–3. Valcu, Valcu, and Kempenaers (2021) defined extra‐pair paternity as the occurrence of paternity outside the social unit, and they excluded data from some cooperatively breeding species, as well as species where individuals do not form pair bonds. The latter included species with female‐only care, lekking species and species that only reproduce via brood parasitism. Valcu, Valcu, and Kempenaers (2021) noted, however, that such species can have clutches sired by multiple males. Our search resulted in seven additional species compared to Valcu, Valcu, and Kempenaers (2021): one cooperative breeder, two species with brood parasitism, two with female‐only care and two socially monogamous species. All these studies have reported the occurrence of broods with mixed paternity (Table A1). We refer to the percentage of broods with mixed paternity as ‘MP broods’ throughout the paper.

The limited number of species for which both data on MP broods and a measure of olfaction was available resulted in 30 species for olfaction measure 1 (diameter; Table 1), 31 species for olfaction measure 2 (volume; Table 2) and 15 species for olfaction measure 3 (OR genes; Table 3). Despite the small numbers of species, all three data sets cover a wide range of orders within the class Aves, representing 13, 11 and 9 orders for measures 1–3, respectively. In total, 58 different species from 15 orders were analysed.

**TABLE 1:** Mixed paternity and olfactory bulb diameter (measure 1). Species of birds, for which we have information on mean percentage of broods showing mixed paternity (MP broods), and the size of olfactory bulb and telencephalon, measured as diameter (mm). Species names are listed alphabetically according to taxonomic order. *N*‐values are given for number of broods, and for brain size (i.e., number of individuals on which olfactory bulb and telencephalon diameter were measured). Values of MP broods are from a supplement to Valcu, Valcu, and Kempenaers (2021) and from references collated in Table A1. For all species but one, diameter measurements of olfactory bulb and telencephalon originate from Bang and Cobb (1968) and Bang (1971). The exception is *Puffinus tenuirostris* , for which both olfactory bulb diameter and brain size values from Warham (1996) are used. Four of the passeriformes (*Poecile atricapillus, Passer domesticus, Serinus canaria, Zonotrichia albicollis*) have fused olfactory bulbs. In those cases, the diameter value given in Bang and Cobb (1968) is for both bulbs. To make those measurements comparable with other taxa, we have halved the values here.

Order	Species name	MP broods (%)	MP broods (*N*)	Olfactory bulb diameter (mm)	Telen‐cephalon diameter (mm)	Brain size (*N*)
Anseriformes	*Anas platyrhynchos*	48	25	4.0	21.0	1
Accipitriformes	*Coragyps atratus*	0.0	16	4.0	24.0	1
Charadriiformes	*Charadrius semipalmatus*	4.2	24	1.5	10.0	1
Charadriiformes	*Fratercula arctica*	0.0	38	2.5	18.0	1
Charadriiformes	*Uria lomvia*	7.4	27	2.7	18.0	1
Coraciiformes	*Colaptes auratus*	1.9	53	1.5	18.0	1
Coraciiformes	*Upupa epops*	13.4	97	2.0	13.6	1
Galliformes	*Meleagris gallopavo*	45.2	31	2.5	18.5	1
Gaviiformes	*Gavia immer*	0.0	47	5.0	25.0	1
Gruiformes	*Fulica americana*	0.0	7	4.0	17.0	2
Gruiformes	*Gallinula chloropus*	0.0	15	3.0	15.0	1
Gruiformes	*Gallinula mortierii*	0.0	6	4.0	15.3	5
Gruiformes	*Porphyrio porphyrio*	0.0	12	4.0	19.0	2
Falconiformes	*Falco peregrinus*	1.9	108	3.4	17.0	2
Passeriformes	*Cinclus cinclus*	5.0	40	1.5	14.0	3
Passeriformes	*Corvus brachyrhynchos*	18.4	87	1.3	26.0	1
Passeriformes	*Hirundo rustica*	40.2	1266	1.5	10.0	2
Passeriformes	*Poecile atricapillus*	29.3	58	0.4	12.5	1
Passeriformes	*Passer domesticus*	27.0	740	0.5	13.0	1
Passeriformes	*Serinus canaria*	0.0	15	0.8	12.5	1
Passeriformes	*Sturnus vulgaris*	34.2	158	1.4	14.5	1
Passeriformes	*Turdus migratorius*	71.9	64	1.2	14.0	1
Passeriformes	*Zonotrichia albicollis*	30.6	412	0.7	14.0	1
Pelecaniformes	*Phalacrocorax carbo*	19.4	98	2.9	20.0	1
Procellariiformes	*Fulmarus glacialis*	0.0	91	5.7	21.0	2
Procellariiformes	*Oceanites oceanicus*	0.0	63	3.6	10.8	4
Procellariiformes	*Oceanodroma leucorhoa*	0.0	48	3.3	10.0	1
Procellariiformes	*Puffinus tenuirostris*	10.8	83	4.9	19.8	4
Sphenisciformes	*Pygoscelis adeliae*	11.1	18	5.0	30.0	1
Strigiformes	*Otus asio*	0.0	23	2.7	18.0	1

**TABLE 2:** Mixed paternity and olfactory bulb volume (measure 2). Species of birds, for which we have information on percentage of broods showing mixed paternity (MP broods), and the size of olfactory bulb and telencephalon, measured as volume (mm^3^). Species names are listed alphabetically according to taxonomic order. *N*‐values are given for number of broods, and for brain size (i.e., number of individuals on which olfactory bulb and telencephalon volume were measured). Values of MP broods are from a supplement to Valcu, Valcu, and Kempenaers (2021) and from references collated in Table A1. Volume measurements of olfactory bulb and telencephalon are taken from Corfield et al. (2015).

Order	Species name	MP broods (%)	MP broods (*N*)	Olfactory bulb volume (mm^3^)	Telen‐cephalon volume (mm^3^)	Brain size (*N*)
Anseriformes	*Anas platyrhynchos*	48.0	25	33.64	4386.68	4
Apodiformes	*Apus apus*	9.5	42	1.36	438.14	1
Charadriiformes	*Sterna hirundo*	2.3	44	4.49	808.53	1
Charadriiformes	*Vanellus chilensis*	18.8	16	6.89	1686.79	1
Coraciiformes	*Dacelo novaeguineae*	0.0	62	5.11	2451.75	1
Galliformes	*Colinus virginianus*	85.3	34	1.11	569.85	1
Galliformes	*Coturnix coturnix*	57.1	21	1.29	394.25	11
Galliformes	*Meleagris gallopavo*	45.2	31	6.36	3269.02	7
Gruiformes	*Porphyrio porphyrio*	0.0	12	35.22	2771.03	3
Passeriformes	*Agelaius phoeniceus*	50.2	968	0.93	635.95	1
Passeriformes	*Baeolophus bicolor*	22.2	9	0.43	534.22	2
Passeriformes	*Carduelis tristis*	26.7	15	0.4	253.08	1
Passeriformes	*Corvus corone*	4.8	62	2	7093.26	12
Passeriformes	*Dumetella carolinensis*	24.8	165	1.48	528.68	1
Passeriformes	*Erythrura gouldiae*	22.8	57	0.37	273.74	1
Passeriformes	*Grallina cyanoleuca*	6.4	47	1.82	1038.98	1
Passeriformes	*Junco hyemalis*	38.2	89	0.83	361.77	3
Passeriformes	*Manorina melanocephala*	5.7	35	1.03	1505.49	1
Passeriformes	*Melospiza melodia*	41.2	966	1.09	467.26	3
Passeriformes	*Passer domesticus*	27.0	740	0.46	608.6	9
Passeriformes	*Passerina cyanea*	48.0	25	1.01	314.79	1
Passeriformes	*Taeniopygia castanotis*	5.7	105	0.13	207.83	1
Passeriformes	*Troglodytes aedon*	35.3	105	0.51	292.89	3
Passeriformes	*Turdus merula*	36.4	77	3.05	1208.87	1
Passeriformes	*Turdus migratorius*	71.9	64	3.05	1135.32	1
Passeriformes	*Zonotrichia albicollis*	30.6	412	1.17	563.8	1
Procellariiformes	*Puffinus tenuirostris*	10.8	83	56.09	2164.22	1
Psittaciformes	*Myiopsitta monachus*	13.9	101	4.44	2733.13	1
Strigiformes	*Athene cunicularia*	1.4	74	15.28	4813.80	1
Strigiformes	*Tyto alba*	1.6	619	8.71	4108.76	1
Struthioniformes	*Struthio camelus*	100	4	66.52	17984.78	1

**TABLE 3:** Mixed paternity and total OR genes (measure 3). Species of birds, for which we have information on percentage of broods with mixed paternity (MP broods), and total number of olfactory receptor (OR) genes. *N*‐values are given for number of broods. Species names are listed alphabetically according to taxonomic order. Values of MP broods are from a supplement to Valcu, Valcu, and Kempenaers (2021) and from references collated in Table A1. For all species, the total number of OR genes originates from Khan et al. (2015), except for *Cyanistes caeruleus* and *Serinus canaria* , whose values are from Steiger et al. (2008). *N* = 1 for each of these estimates. Body size (average body mass, g) is from Dunning Jr (2008). We pooled the sexes when data were available for each sex separately, and for polytypic species we selected the body mass values for the subspecies that was used for determining the number of OR genes (see Khan et al. 2015 and references therein).

Order	Species name	MP broods (%)	MP broods (*N*)	Total number of OR genes	Body size (g)
Anseriformes	*Anas platyrhynchos*	48.0	65	344	1171
Falconiformes	*Falco peregrinus*	1.9	108	460	610
Falconiformes	*Haliaeetus albicilla*	0.0	50	283	4793
Galliformes	*Meleagris gallopavo*	45.2	31	313	6050
Passeriformes	*Acanthisitta chloris*	0.0	65	222	7.0
Passeriformes	*Corvus brachyrhynchos*	18.4	87	229	506
Passeriformes	*Cyanistes caeruleus*	47.0	2947	218	10.6
Passeriformes	*Geospiza fortis*	35.5	93	182	24.0
Passeriformes	*Serinus canaria*	0.0	15	166	24.3
Passeriformes	*Taeniopygia castanotis*	5.7	105	688	12.1
Pelecaniformes	*Phalacrocorax carbo*	19.4	98	270	2110
Procellariiformes	*Fulmarus glacialis*	0.0	91	370	613
Sphenisciformes	*Pygoscelis adeliae*	11.1	18	320	4850
Strigiformes	*Tyto alba*	1.6	619	321	520
Struthioniformes	*Struthio camelus*	100.0	4	318	111000

Estimates of the total number of OR genes in birds are available from six papers (Steiger et al. 2008; Wang et al. 2013; Khan et al. 2015; Vandewege et al. 2016; Yohe et al. 2020; Driver and Balakrishnan 2021). Seven species are reported in two or more of these papers, allowing a comparison between studies (Table A2). As can be seen from that table, the estimates vary markedly within species. The most recent publications (Yohe et al. 2020; Driver and Balakrishnan 2021) show substantially lower numbers than the previous ones for all seven species. We have chosen to use the estimates by Khan et al. (2015) in our study, as this paper by far covers the greatest number of species (48 species of birds, based on whole genome sequencing originally compiled by Jarvis et al. 2014), compared to the other studies (eight species in Steiger et al. 2008; two species in Wang et al. 2013 and Vandewege et al. 2016; eight species in Yohe et al. 2020; five species in Driver and Balakrishnan 2021). Khan et al. (2015) also contains the greatest number of species for which we also have data on mixed paternity (13 species). We used the estimates by Steiger et al. (2008) for two additional species that were not covered by Khan et al. (2015), resulting in 15 species in total. We found no new matches between the species with mixed paternity data and the species included in Wang et al. (2013), Vandewege et al. (2016), Yohe et al. (2020) or Driver and Balakrishnan (2021) that were not already covered by Steiger et al. (2008) or Khan et al. (2015), thus allowing us to evade potential problems associated with using markedly smaller estimates of total number of OR genes for a subset of species.

Given the small numbers of species for which we have both a measure of olfaction and of mating pattern, and that less than half of these species are found in the most comprehensive molecularly based phylogenetic trees that are available to date (Jetz et al. 2012; Feng et al. 2020), we have refrained from using phylogenetically controlled analyses, as originally intended since most of the inter‐specific variation in mixed paternity among birds is located at the taxonomic family level or above (Arnold and Owens 2002). Instead, we have chosen an exploratory approach, examining the correlations between the measures of mixed paternity (MP broods) and olfaction (olfactory bulb diameter, olfactory bulb volume or total number of OR genes) using the species data. Given our relatively small sample sizes, we decided to use non‐parametric statistics (Conover 1980). Using parametric methods, however, yielded qualitatively similar results (not shown).

Body size varies between phylogenetically different groups, and it is positively correlated with brain size, affecting both telencephalon and olfactory bulb size. As expected, telencephalon diameter correlated positively with olfactory bulb diameter (Spearman rank correlation, rho = 0.58, *N* = 31, *p* < 0.001). Likewise, telencephalon volume correlated positively with olfactory bulb volume (Spearman rank correlation, rho = 0.85, *N* = 32, *p* < 0.001). Therefore, to control for telencephalon size (and indirectly also body size), we used partial correlation analyses, when correlating olfactory bulb diameter with MP broods, and olfactory bulb volume with MP broods. However, we used a bivariate correlation analysis to correlate total number of OR genes with MP broods, since the total number of OR genes was not associated with body mass (values sourced from Dunning Jr 2008; Spearman rank correlation, rho = 0.33, *p* = 0.24, *N* = 15; Table 3), and hence did not indicate a need to control for body size in this analysis.

## 3 Results

**FIGURE 1:**
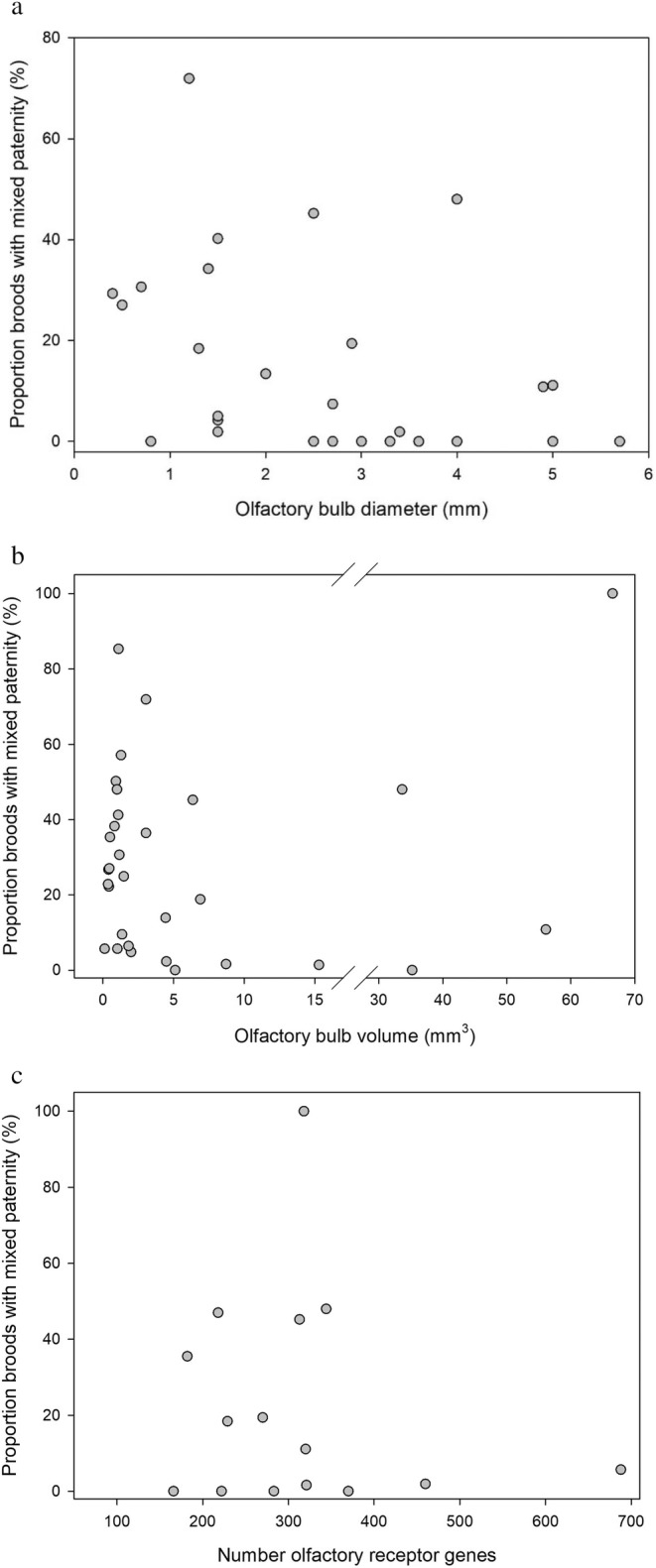
Relationship between percentage of broods with mixed paternity and (a) olfactory bulb diameter, (b) olfactory bulb volume, (c) total number of olfactory receptor genes. The graphs show actual values for descriptive purposes, but in (a) and (b), the relationships were tested in partial correlations, controlling for brain (telencephalon) size. See Tables 1, 2, 3 for species and orders included in the analyses.

We found a negative correlation between olfactory bulb diameter and MP broods (partial correlation, controlling for telencephalon diameter: rho = −0.56, df = 27, *p* < 0.002; Figure 1a and Table 1). However, we found no significant correlation between olfactory bulb volume and MP broods (partial correlation, controlling for telencephalon volume: rho = 0.013, df = 28, *p* = 0.95; Figure 1b and Table 2). The data point in the upper right corner in Figure 1b, representing the ostrich, *Struthio camelus* , appears to be an outlier. However, the correlation between paternity and bulb volume is non‐significant also when excluding this data point (partial correlation, controlling for telencephalon volume: rho = −0.027, df = 27, *p* = 0.89). Finally, the total number of OR genes did not correlate significantly with MP broods (Spearman's correlation: rho = −0.031, df = 15, *p* = 0.91; Figure 1c and Table 3).

## 4 Discussion

We found that species of birds that have small olfactory bulbs for their brain size, had larger percentage of broods sired by multiple males and *vice versa*. These results were true in the data available to us for olfactory bulb size measured as diameter, whereas another data set in which olfactory bulb size was measured as volume, did not show any correlation. Our third estimate of olfaction, namely total number of OR genes, did not correlate with percentage of broods sired by multiple males.

Our finding that olfactory bulb diameter correlates negatively with the percentage of broods with mixed paternity fits with our prediction that birds that have access to a highly developed olfaction would be better at finding a good match at the stage of pair formation, compared to birds with less developed olfaction. These results are based on data from only 30 species, analysed without phylogenetic correction, and should be seen as preliminary findings. Still, our data represent 13 different orders of birds, which thus suggests that these general patterns may hold at a broad phylogenetic scale. Whether they are of importance also among more closely related species remains to be tested.

Our results are consistent with the idea suggested by Griffith and Immler (2009) that the high levels of mixed paternity found in passerines may be due to a reduced ability to evaluate the genetic profile of their mate against their own profile before mating. Although recent research shows that some passerines are able to evaluate MHC similarity and diversity based on olfaction (Grieves et al. 2019), that ability may still be poorer compared to other taxa. Across birds, high rates of mixed paternity are associated with high adult mortality rates (Arnold and Owens 2002). Since passerines typically show shorter life spans than most non‐passerines (e.g., Wasser and Sherman 2010), engaging in extra‐pair copulations might be a better option to compensate for a potential suboptimal social mate choice than divorce in these species (but see Cezilly and Nager 1995).

Intriguingly, we did not find a correlation between olfactory bulb volume and percentage of broods with mixed paternity. The volume‐based data represent a very different set of species, compared to the diameter‐based data discussed above, but the sample size of 31 species was similar, from 11 different orders. The species that shows the highest frequency of mixed paternity broods (100%) is the ostrich, *S. camelus* , which has a large olfactory bulb volume (67 mm^3^). This species has a complex communal breeding system that includes polygamous matings by both sexes (Kimwele and Graves 2003). The ostrich is also the largest living bird, but that should not have affected our results since we controlled for the influence of body size, by controlling for brain size in the analyses. Furthermore, excluding this data point from the analysis still yields a non‐significant correlation between paternity and bulb volume.

The total number of olfactory receptor genes did not correlate significantly with the percentage of broods with mixed paternity. However, this was our smallest data set, consisting of only 15 species of birds from 9 orders. It was compiled from two different studies (Steiger et al. 2008; Khan et al. 2015), which made the estimated total number of OR genes less comparable between species than would have been ideal. With larger data sets, collected using a consistent sequencing approach, it is possible that also OR genes will be found to correlate with mixed paternity. Indeed, the two species with highest and lowest values of mixed paternity, the ostrich, *S. camelus* (100% mixed paternity broods and 318 OR genes) and zebra finch, *Taeniopygia castanotis* (6% mixed paternity broods and 688 OR genes), fit a pattern that OR genes may correlate negatively with mixed paternity. On the other hand, OR gene evolution has been shown to be decoupled from olfactory bulb evolution among tetrapods (Yohe et al. 2020), which may explain the difference in results between olfactory bulb diameter and total number of OR genes.

We are aware of the limitations of our study (small number of species, correlative data, no phylogenetic analysis). Still, by offering this preliminary exploration, we want to inspire and encourage others to collect more data for a full‐scale test of the hypothesis in the future. To efficiently extend the list of species, collection of olfactory measures and OR gene sequencing should target species of birds for which parentage data already exist (Valcu, Valcu, and Kempenaers 2021), as phylogenetically broad as possible, and with a particular focus on non‐passerines that are clearly under‐represented in the current data sets. The three olfactory measures used were chosen because data exist on them. However, a stringent test of the underlying assumptions that they accurately reflect olfactory ability would be crucial, and new measures may need to be developed, for example, based on a standardised behavioural assay. Existing estimates of total number of OR genes show remarkable differences between different studies, likely reflecting differences in methodology (Table A2). This highlights the importance of using a uniform method (including sequencing and bioinformatic pipeline) for all studied species. Finally, since both extra‐pair paternity (Arnold and Owens 2002; Brouwer and Griffith 2019) and relative olfactory bulb size (Corfield et al. 2015) show clear impact of phylogeny, phylogenetically controlled analyses will be needed in future research.

At the outset of our study, we assumed that olfaction has first evolved for other purposes, such as foraging, and that mating pattern, reflected in degree of mixed paternity, has evolved as a result. With the current data, we cannot determine if this is the case, and therefore, we have analysed all our data using correlations. In a future phylogenetically based study, it would be essential to establish if our assumption is true, that is, whether olfaction evolved first, and thus may be considered a cause and mating pattern a consequence, or if it may be the other way around, with mating pattern influencing olfactory ability.

In conclusion, our findings that bird species that have large olfactory bulbs for their brain sizes had smaller percentage of broods sired by multiple males, and *vice versa*, provides tentative support for our prediction that birds with highly developed olfaction would be better at finding a well‐matched mate at the stage of pair formation compared to birds with less developed olfaction. If this interesting pattern holds for a larger data set, tested using phylogenetic analysis, it will substantially improve our understanding of the pair formation process, and of underlying physiological constraints, affecting mating systems in birds.

## Author Contributions

**Charlotta Kvarnemo:** conceptualization (lead), data curation (lead), formal analysis (supporting), investigation (equal), methodology (equal), supervision (equal), writing – original draft (lead), writing – review and editing (equal). **Alice Anderstedt:** data curation (equal), formal analysis (supporting), investigation (supporting), writing – review and editing (supporting). **Maria Strandh:** conceptualization (equal), data curation (equal), investigation (equal), methodology (supporting), writing – review and editing (supporting). **Donald Blomqvist:** conceptualization (equal), data curation (equal), formal analysis (lead), investigation (equal), methodology (equal), supervision (equal), writing – original draft (equal), writing – review and editing (equal).

## Conflicts of Interest

The authors declare no conflicts of interest.

## Data Availability

All data are available in Tables 1, 2, 3. Additional background information is included in the Appendix 1.

## APPENDIX 1

**TABLE A1:** Mean percentage broods with mixed paternity (MP) for seven species that were not included in Valcu, Valcu, and Kempenaers (2021); see main text for further details. Breeding system denotes the social mating and parental care system. BC = biparental care, CBP = conspecific brood parasitism, FC = female care. Species names are listed alphabetically according to taxonomic order. *N*‐value shows number of broods that were analysed, and references refer to the original publications.

Order	Species name	Breeding system	MP broods (%)	MP broods (*N*)	References
Charadriiformes	*Vanellus chilensis*	Cooperative	18.8	16	Saracura, Macedo, and Blomqvist (2008)
Galliformes	*Coturnix coturnix*	Polygamy, FC	57.1	21	Sanchez‐Donoso et al. (2018)
Galliformes	*Meleagris gallopavo*	Promiscuous, FC	45.2	31	Krakauer (2008)
Gruiformes	*Fulica americana*	Monogamy, BC, CBP	0.0	7	Lyon, Hochachka, and Eadie (2002)
Gruiformes	*Gallinula chloropus*	Monogamy, BC, CBP	0.0	19	McRae and Burke (1996)
Passeriformes	*Poecile atricapillus*	Monogamy, BC	29.3	58	Otter et al. (1998)
Strigiformes	*Otus asio*	Monogamy, BC	0.0	23	Lawless et al. (1997)

**TABLE A2:** Estimates of total number of OR genes differ markedly between studies. The table shows total number of OR genes estimated in Steiger et al. (2008), Wang et al. (2013), Khan et al. (2015), Vandewege et al. (2016), Yohe et al. (2020) and Driver and Balakrishnan (2021), for seven species of birds that are included in more than one study. Species for which we have access to data on mixed paternity, and hence that are part of our study, are underlined. Sequencing methods (in most cases sourced from NCBI; https://www.ncbi.nlm.nih.gov/datasets/genome/) are indicated.

Order	Species name	Steiger et al. (2008)	Wang et al. (2013)	Khan et al. (2015) sequenced by Jarvis et al. (2014)	Vandewege et al. (2016)	Yohe et al. (2020)	Driver and Balakrishnan (2021)
Anseriformes	*Anas platyrhynchos*	430^a^		344^d^			
Galliformes	*Gallus gallus*	638^a^	433^b^	674^a^	522^a^	69^c^	355^c^, 272^a^
Gaviiformes	*Gavia stellata*			369^b^		69^b^	
Passeriformes	*Manacus vitellinus*			227^b^			117^c^, 9^b^
Passeriformes	*Taeniopygia castanotis*		544^b^	688^a^	541^a^	11^c^	69^c^, 184^a^
Pelecaniformes	*Phalacrocorax carbo*			270^b^		28^b^	
Trochiliformes	*Calypte anna*			324^b^			109^c^, 27^b^

^a^ Sanger.

^b^ Illumina HiSeq.

^c^ PacBio (alone or in combination with other methods).

^d^ Solexa.
